# Apple pectin-based *Zataria multiflora* essential oil (ZEO) nanoemulsion: An approach to enhance ZEO DNA damage induction in breast cancer cells as *in vitro* and *in silico* studies reveal

**DOI:** 10.3389/fphar.2022.946161

**Published:** 2022-09-05

**Authors:** Fahimeh Salehi, Hossein Behboudi, Elaheh Salehi, Sussan K. Ardestani, Firoozeh Piroozmand, Gholamreza Kavoosi

**Affiliations:** ^1^ Institute of Biochemistry and Biophysics, Department of Biochemistry, University of Tehran, Tehran, Iran; ^2^ Faculty of Biology, Medicinal Plants and Drugs Research Institute, Shahid Beheshti University, Tehran, Iran; ^3^ Tehran Medical sciences, Islamic Azad University, Tehran, Iran; ^4^ Department of Microbiology, College of Science, University of Tehran, Tehran, Iran; ^5^ Institute of Biotechnology, Shiraz University, Shiraz, Iran

**Keywords:** apple pectin, *Zataria multiflora* essential oil, nanoemulsion, breast cancer, apoptosis, DNA damage

## Abstract

*Zataria multiflora* essential oil (ZEO) is a natural complex of compounds with a high apoptotic potential against breast cancer cells and minor toxicity toward normal cells; however, similar to many essential oils, ZEO utilization in pharmaceutical industries has limitations due to its labile and sensitive ingredients. Nanoemulsification based on natural polymers is one approach to overcome this issue. In this study, an apple pectin-ZEO nanoemulsion (AP-ZEONE) was prepared and its morphology, FTIR spectra, and physical properties were characterized. Furthermore, it was shown that AP-ZEONE substantially suppresses the viability of MDA-MB-231, T47D, and MCF-7 breast cancer cells. AP-ZEONE significantly induced apoptotic morphological alterations and DNA fragmentation as confirmed by fluorescent staining and TUNEL assay. Moreover, AP-ZEONE induced apoptosis in MDA-MB-231 cells by loss of mitochondrial membrane potential (ΔΨm) associated with the accumulation of reactive oxygen species (ROS), G2/M cell cycle arrest, and DNA strand breakage as flow cytometry, DNA oxidation, and comet assay analysis revealed, respectively. Spectroscopic and computational studies also confirmed that AP-ZEONE interacts with genomic DNA in a minor groove/partial intercalation binding mode. This study demonstrated the successful inhibitory effect of AP-ZEONE on metastatic breast cancer cells, which may be beneficial in the therapy process.

## 1 Introduction

Breast cancer, a highly heterogeneous disease in terms of histology, response to treatment, and patterns of metastasis, is the most prevalent cause of cancer death among women (Hati et al., 2016). Despite the advancements in identifying the chemotherapy options for cancer, the success of its treatment has been very limited due to the wide range of adverse effects of chemical drugs that lead to high mortality rates. Therefore, looking through natural compounds as a rich source of safer drugs to treat cancer is reasonable. Plant-derived compounds with the ability to induce apoptosis via various signaling pathways have been suggested as an alternative approach in the treatment of cancer. Many of these agents cause toxicity through covalent or non-polar binding to the DNA molecule (Jamali et al., 2018). Etoposide (ETO) (topoisomerase II inhibitor) and topotecan (topoisomerase I inhibitor) with diverse clinical applications in cancer therapy are in this family of compounds (Mann, 2002; Salehi et al., 2017). *Zataria multiflora* essential oil belongs to the carvacrol/thymol chemotype and is the endemic herb of Iran, Afghanistan, and Pakistan often used in Iranian traditional medicine to treat abdominal pain, inflammation, asthma, and cancer symptoms. Previous studies have described the antioxidant, antifungal, and antimicrobial activities of the essential oil extracted from *Z. multiflora* (Saei-Dehkordi et al., 2010; Ghasemi Pirbalouti et al., 2011; Sajed et al., 2013). Recently, our studies revealed the involvement of *Z. multiflora* essential oil in ROS-mediated mitochondrial membrane depolarization, DNA damage/fragmentation, caspases activity, and cell cycle arrest, which eventually lead to apoptosis in breast cancer cells; however, it did not affect normal cells (Salehi et al., 2017). Similar to many essential oils, the utilization of ZEO in pharmaceutical industries has limitations due to its volatility, sensitivity to oxidation, light, heat, and low dispersibility in hydrophilic conditions (Ali et al., 2020). One approach to overcome these issues is to nanoemulsify such essential oils in natural polymers. These biopolymers have favorable properties such as biodegradability, biocompatibility, antimicrobial activity, delay in oxidation, and usability as carriers of many natural compounds such as essential oils (Kurtz and Lawson, 2019; Salehi et al., 2020).

The aforementioned pieces of evidence provided a strong motivation to investigate the effect of Nanoemulsification on the anticancer activity of ZEO incorporated into apple pectin (AP) through mild emulsification. The cytotoxic effect of AP-ZEONE on breast cancer cell lines MDA-MB-231, T47D, and MCF-7 were evaluated. L929 fibroblast cells were used as controls to assess the selectivity of the AP-ZEONE. Further, we demonstrated that AP-ZEONE exposure induces apoptosis in MDA-MB-231 cells via the mitochondrial pathway. Moreover, the biological activity of the AP-ZEONE was examined *in vitro*, concerning their interaction mode with double-strand DNA (dsDNA) by applying spectroscopic approaches and computational simulations to elucidate the molecular mechanisms of ZEO-DNA binding. Molecular docking was performed for the five main constituents of ZEO against DNA double-helix.

## 2 Materials and methods

### 2.1 Preparation and characterization of ZEO

The aerial parts of Z. *multiflora* (herbarium number 24985) were collected from the mountainous areas of Marvdasht town, Iran (Kavoosi and Rabiei, 2015). The identification of collected plants was thankfully carried out by Professor Ahmad Reza Khosravi, plant taxonomist at the Biology Department, Shiraz University, Iran. The plant materials were dried in the shade for 3–4 days. The air-dried plant samples (100 g) were hydro-distilled for 3 h using the Clevenger apparatus to collect the essential oils. Gas Chromatography-Mass Spectrometry (GC-MS) was carried out using an Agilent Gas Chromatography (Agilent 7890B GC 7955AMSD) coupled with a single quadrupole mass spectrometer and silica HP5MS column (30 m × 0.25 mm × 0.25 µm) as described previously. The temperatures of the ion source and that of the interface were 210 and 270°C, respectively. The programmed temperatures of the oven were as follows: 4 min at 60°C, rising to 140°C at 20°C/min, then increased to 220°C at 10°C/min, and finally, fixed for 10 min at 220°C. Mass spectra were obtained at 80 eV with mass ranges of 50–400 m/z. The essential oil compositions were determined by comparing the fragmentation patterns of the peaks with libraries’ mass spectra (Wiley 7n and NISTO5A) (Salehi et al., 2017).

### 2.2 Preparation and characterization of ZEO emulsion

To prepare ZEO emulsion, 1 ml (equal to 1,000 mg, ZEO density ≃ 1 g/cm^3^) of ZEO was added to 100 ml of distilled water. Polysorbate-20 (10 mg/ml) was then added to the mix and incubated at 35°C for 2 days until a milky solution (emulsion) was obtained. In order to standardize ZEO, total phenol content was determined by Folin–Ciocalteu reagent using gallic acid as standard (Ainsworth and Gillespie, 2007). In brief, 200 µl of emulsion or standard gallic acid solutions (0–500 µg) were added to 1 ml Folin–Ciocalteu reagent (10%) plus 0.3 ml Na_2_CO_3_ (10%). The mixture was left in the dark at ambient temperature for 60 min. The absorbance of the solution was read at 765 nm. The concentration of phenolic contents was determined using the equation calculated from the gallic acid standard curve (y = 0.0946x–0.997; R2 = 0.994). For *in vitro* or cell culture analysis ZEO emulsion was diluted in the medium culture at a concentration of 10 mg/ml gallic acid equivalent. For *in vitro* or physical analysis, ZEO emulsion was diluted in distilled water at a concentration of 10 mg/ml gallic acid equivalent.

### 2.3 Preparation and characterization of pectin-tween-ZEO dispersion

A homogeneous solution containing apple pectin (1 g) in distilled water (80 ml) was prepared and stirred continuously at room temperature. Pectin dispersion incorporated with ZEO emulsion (Tween20 (100 μg/ml) was used as emulsifier) was prepared by mixing the ZEO emulsion (100 mg gallic acid equivalent per Gram of pectin equal to 1,000 μg/ml of pectin dispersion) with the pectin solution and stirring it for complete mixing, at room temperature. In the end, the final volume was adjusted to 100 ml using distilled water and the final dispersions were contributed to physical analysis. It should be noted that AP-tween-ZEO dispersion was stable for 120 days at 4°C.

The electrophoretic mobility/zeta potential of the particles and effective hydrodynamic diameter were obtained based on the Phase Analysis Light Scattering (PALS) and Dynamic Light Scattering (DLS) techniques, respectively, using Brookhaven Instrument Corporation 90 Plus particle size analyzer (New York, USA). The Bi-9000 particle sizing software was used to analyze the polydispersity and the average effective hydrodynamic diameter. The Bi-PALS zeta-potential analyzer software provides a measure of zeta-potential and an average of electrophoretic mobility by the Smolouchewsky model. The surface tension of the composite solution was calculated by the Du Nouy tensiometer (Kruss, Germany) at room temperature. The viscosities of the composite solutions were quantified using the MCR302 rheometer (Anton Paar) at a shear rate of 0.01–100 s^−1^. The value at the shear rate of 10 s^−1^ was reported. Fourier Transform Infrared (FTIR) spectroscopy of the samples was performed by means of Bruker FTIR (Germany) in the range between 4,000 and 400 cm^−1^.

### 2.4 Preparation of pectin-essential oil nanoparticles for Scanning Electron Microscopy (SEM)

A dilute solution of apple pectin-essential oil nanoemulsion was applied to a 3 ml glass syringe. The nozzle was a gauge 20 blunt-ended stainless steel hypodermic needle. The screen collector was a sheet of aluminum. A gamma high voltage DC power supply was connected to the needle as the emitting electrode of positive polarity. The grounding electrode was attached to the aluminum sheet collector. Under a fixed 10kV/10 cm electrostatic field strength and 10 min collection time, pectin-essential oil nanoemulsion was electrosprayed with a feed rate of 0.3 ml/h After gold coating the magnified images of the nanoparticles were taken by a scanning electron microscope (Tescan-vega3, Czech Republic). SEM images were taken with ×1000 magnification under 18 kV accelerating voltage.

### 2.5 Cell culture and treatment

MCF.7 (ATCC No. HTB-22), highly invasive MDA. MB.231 (ATCC No. HTB-26), epithelial T47D (ATCC No. HTB-133) human breast cancer cell lines, and normal L929 (ATCC No. CCL-1) mouse fibroblast cells were procured from the National Cell Bank of Iran (Pasteur Institute, Iran) and were cultured in RPMI 1640 medium complemented with 10% fetal bovine serum, 100 μg/ml streptomycin and 100 U/ml penicillin in an incubator (5% CO_2_ in air at 37°C). Then, the cells were collected for all the experiments. RPMI was used to dilute the AP polymer, ZEO, and AP-ZEONE stock solutions to proper concentrations. After the treatment with AP-ZEONE for 24h, MDA. MB.231, T47D, and MCF.7 cells were observed under an inverted microscope (Ax overt 25, Zeiss, Germany) to recognize the morphological variations as compared to the control samples.

### 2.6 Anti-proliferation assay

Cell viability of the test materials was determined by measuring the formazan production in microplates using MTT Assay. MCF.7, MDA. MB.231, T47D breast cancer cells, and L929 normal fibroblast cells were seeded on 96-well plates (1.4 × 10^4^/well) followed by 24 h incubation. Then, cells were exposed to a serially diluted concentration of AP-ZEONE (0–125 μg/ml) for another 24, 48, and 72 h at 37°C, 5% CO_2_. 200µl/well of MTT [3-(4, 5-dimethylthiazol-2-yl)-2, 5-diphenyltetrazolium Bromide] reagent (Sigma-Aldrich) (0.5 mg/ml in PBS) was then added and further incubated for 4 h. Finally, 100 µl dimethyl sulfoxide (DMSO) was dispensed into each well to dissolve formazan crystals. The amount of formazan production was measured at 492 nm in an ELISA reader (wave xs2, BioTek, USA) and is associated with the number of living cells. The inhibitory rates and concentration of the test material that killed 50% of the cells (IC50 value) were then calculated in Excel (Microsoft).

### 2.7 Apoptotic detection

#### 2.7.1 Acridine orange/ethidium bromide (AO/EB) staining

The apoptotic or necrotic morphology of the cells treated with AP-ZEONE was detected using AO/EB staining. 3.5 × 10^5^ cells/well were grown in 6-well plates overnight followed by the AP-ZEONE treatment at IC50 concentration. Then, the cells were washed and stained with AO/EB, and the morphologic properties of the cells were observed under a fluorescence microscope (Axioskop 2plus, Zeiss, Germany).

#### 2.7.2 Annexin V-PI staining

Induction of apoptosis in MDA. MB.231 by AP-ZEONE was quantified by flow cytometry analysis after staining cells with annexin V-FITC/PI kit (BioVision) according to the manufacturer’s instruction. In brief, cells (3.5 × 10^5^/well) were seeded in 6-well plates overnight and then were treated with 8 μg/ml of AP-ZEONE (MDA.MB.231 cells IC50) for 4 h. Subsequently, cells were trypsinized and washed with PBS, stained with annexin V-FITC and PI to detect early and late apoptosis. Cell analyses were performed on a FACS machine (Partec PAS, Germany).

### 2.8 DNA damage studies

#### 2.8.1 DNA fragmentation and comet assay

To quantitatively evaluate the apoptosis, a DNA degradation assay was performed by electrophoresis on an agarose gel and Terminal deoxynucleotidyl transferase dUTP nick end labeling (TUNEL) assay as reported earlier (Salehi et al., 2017). In brief, for DNA laddering pattern, following the treatment with appropriate concentrations of AP-ZEONE (4 and 8 μg/ml) for 7 h, MDA. MB.231 and T47D cells were collected, washed twice with PBS, and DNA extraction was performed by the usual standard technique. Subsequently, DNA samples were dissolved in TE buffer. Samples were run on 2% agarose gel for 2 h and visualized with EB staining. Treated cells were stained with Tunnel assay *in situ* cell death detection kit, Fluorescein (Roche), according to the manufacturer’s instruction. In brief, after treatment (4 h), fixation and permeabilization of the cells were performed using a mixture of 4% paraformaldehyde solution, 1% Triton X-100 and 0.1% sodium citrate. Cells were labeled with a TUNEL reaction mixture at 37°C for an additional 60 min. The percentage of TUNEL-positive cells was measured via flow cytometry. To further confirm the DNA damage at the single-cell level, the alkaline comet assay to detect single and double-strand breaks was performed according to the protocol described previously (Salehi et al., 2017). Briefly, following the treatment with IC50 of AP-ZEONE for 2h, collected cells were mixed with low-melting-agarose at 37°C and then poured on a glass slide previously coated with normal-melting-agarose and covered with a coverslip to cast a very thin layer agarose gel. Casted slides were immersed in lysis buffer (100 mM EDTA, 2.5 M NaCl, 10 mM Tris-base, 1% Triton X- 100 pH 10 in 4°C) for 1 h then electrophoresed in lysis buffer for 30 min at 0.6 V/cm, neutralized and stained with SYBR green for 15 min. In the end, comets were visualized by a fluorescent microscope (Axioscope two plus, Zeiss, Germany), and analyzed in Open Comet software.

#### 2.8.2 DNA oxidation analysis

MDA-MB-231 cells were first treated with 2,4,8 ug/ml of AP-ZEONE for 2 h. Subsequently, their genomic DNA was extracted along with untreated control samples by standard phenol-chloroform extraction protocol. 5 μg of each DNA sample was then digested with nuclease P1 (sigma N8630) and alkaline phosphatase (NEB M0290S) to increase sample sensitivity to 8-oxo detection Elisa kit (Cayman chemicals, 589,320). The test was performed according to ELISA manufacturer instruction and the concentration of 8-oxo in each sample was expressed as pgml^−1^.

### 2.9 ROS detection assay

Intracellular production of ROS in AP-ZEONE treated cells was measured using 2′-7’dichlorofluorescin diacetate (DCFH-DA) (Sigma-Aldrich, USA). To perform this assay, 1 × 10^5^ MDA. MB.231 cells/well were seeded for 24 h. Cells were then exposed to AP-ZEONE IC50 for 12 h. Subsequently, cells were collected washed (with PBS), and resuspended in DCFH-DA working solution (20 µM) at 37°C for 30 min in the dark. Stained cells were washed with PBS and intracellular Dichlorofluorescein (DCF) fluorescence was recorded by flow cytometry with excitation at 485–495 nm and emission at 525–530 nm.

### 2.10 Determination of mitochondrial membrane potential (MMP)

MDA.MB.231 cells seeded for 24 h were treated with AP-ZEONE IC50 for 12 h and then incubated with 400 µl 50 µM Rhodamine 123 for 30 min at 37°C. Cells were then harvested and washed with pre-warmed PBS and the fluorescent intensity of the dye was measured with excitation and emission at 488 and 525–530 nm on a flow cytometer, respectively. The fluorescence intensity of Rhodamine 123 represented the cellular levels MMP.

### 2.11 Cell-cycle analysis using flow cytometry

Cell-cycle analysis was performed by determining the DNA content in treated and untreated cells using flow cytometry following propidium iodide (PI) staining. 3.5 × 10^5^ MDA. MB.231 cells/well were cultured and treated with 4 and 8 μg/ml of AP-ZEONE for 4 and 24 h, subsequently, cells were trypsinized, collected, and washed with cold PBS twice. Samples were then fixed with ice-cold 70% ethanol at 4°C overnight. Finally, fixed cells were washed and resuspended in cold PBS containing 20 μg/ml PI and 20 μg/ml RNase and incubated at 37°C for 30 min. PI fluorescence was recorded by flow cytometer and analyzed by FlowJo software.

### 2.12 DNA binding studies

#### 2.12.1 Reagents and UV–visible spectroscopy

Rat Hepatocyte DNA (RH-DNA) was extracted by using the common and standard phenol-chloroform method and its concentration and quality were determined at 260 nm by Nanodrop (Thermo Fisher Scientific-USA) and UV-visible spectrophotometer (Cary 100 Bio-model, Australia). The liver specimens were obtained in accordance with guidelines of the Ethical Committee of the Pasteur Institute of Iran (ECPII) and the National Institutes of Health guide for the care and use of laboratory animals (NIH Publications NO. 8023, revised 1978). The purity of DNA was monitored by assessing the absorbance ratio of the A260nm/A280 nm. The samples with A260nm/A280 nm > 1.8 were accepted as pure DNA. To confirm the integrity of extracted DNA, electrophoresis was performed on 0.8% agarose gel. The DNA solution was prepared in phosphate (Na_2_HPO_4_-NaH_2_PO_4_, 0.1 M) buffer and the pH was adjusted to 7.4 and stored at 20°C in dark. UV–visible spectra were recorded on Cary 100 Bio-model, Australia. Spectra of AP-ZEONE and AP-ZEONE–DNA complexes were scanned in the wavelength range of 190–600 nm. An increasing concentration of AP-ZEONE was titrated against 50 μg/ml of DNA. The phosphate buffer was considered blank.

#### 2.12.2 Fluorescence measurements

Fluorescence detection was carried out using a Cary Eclipse spectrophotometer (Australia) by maintaining the DNA concentration (50 μg/ml) constant and varying the AP-ZEONE concentration (0–1.9 × 10^–3^ M) using 1.0 cm quartz cells. EB displacement assay was performed with a concentration of 2.6 µM. Excitation was set at 500 nm and changes in the emission spectra were recorded in the range of 530–700 nm after both excitation and emission slits were set to 10 nm. 0.1 M phosphate buffer (pH 7.4) was used in all experiments.

#### 2.12.3 CD spectroscopy

The changes of Circular Dichroism (CD) spectra related to both DNA and AP-ZEONE alone and AP-ZEONE-DNA complex were collected at a wavelength from 200 to 400 nm on CD 215, Aviv, USA, spectrophotometer by quartz cell path (with the length of 1.0 cm). Additionally, the respective blanks were subtracted from each curve. The concentration of DNA and AP-ZEONE was kept at 100 and 8 μg/ml, respectively.

### 2.13 Computational study of ZEO interaction with the DNA molecule

A B-DNA 3D structure (PDB ID: 1BNA) was considered as the receptor molecule to study the probability and mode of interaction of DNA and ZEO. The interaction models of carvacrol, g-Terpinene, carvacrol methyl ether, p-Cymene, and thymol (the five prominent ingredients of ZEO as previously analyzed by GC-MS) were studied using Autodock 4.2.100. Conformers were built for each docking study and an optimal conformer was selected as the most probable mode of interaction. Docking results were analyzed using LigPlot^+^ software.

### 2.14 Statistical analysis

The values for all experimental data are expressed as mean ± SD (at least three individual experiments). Microsoft Excel 2013 software was used to analyze data and statistically significant values are considered *p* < 0.05 by the *t*-test.

## 3 Results

### 3.1 Characterization of AP-ZEO nanoemulsion

Mild emulsification conditions have been used in this study to prepare AP-polysorbate-ZEO dispersion. The zeta-potential, particle size, surface tension, and viscosity are key parameters for particle stability measured to determine the tendency of particles for aggregation or dispersion in biological fluids as well as the ability to deliver the payload. The rheological properties of polysorbate, polysorbate-ZEO, pectin, pectin-polysorbate, and pectin-polysorbate-ZEO dispersion are summarized in Table 1. As indicated in Table 1, pectin solution displayed higher surface tension, zeta potential, particle size, and viscosity rather than pectin-polysorbate and pectin-polysorbate-ZEO dispersion. This means that the addition of ZEO at the applied concentration led to a slight decrease in the particle size, zeta potential, surface tension, and viscosity of the pectin particles. In AP dispersion, the interaction between pectin chains (via van der Waals interactions and hydrogen bonds) made a pectin particle with an average size of 220 nm. In pectin-polysorbate and pectin-polysorbate-ZEO dispersion, polysorbate or ZEO can interact with pectin through van der Waals attractive forces, so the average particle sizes of pectin and related zeta potential values decrease. In this range of concentration, the pectin chain-chain interactions were substituted with pectin chain-polysorbate or pectin chain-ZEO interactions. Therefore, pectin chains are detached from each other and the average particle size decreases. The zeta potential for AP dispersion was largely negative (Table 1) due to the negative charge of carboxyl (COOH) groups of galacturonic acid residues. If the particle charge increases, the electrostatic repulsion also increases while particle aggregation tends to be reduced. In our experiments both particle size and zeta-potential decreased with the addition of ZEO which led to a larger specific surface area of the nanoparticles as would be expected for smaller particles. The reduction of zeta potential could be related to the hided pectin charge by polysorbate-ZEO droplets. The addition of polysorbate-ZEO led to a slight decrease in the surface tension of the pectin particle. This decrease can be attributed to the surfactant (detergent) activity of polysorbate or to some extent lipophilic nature of the ZEO. The surface tension of a liquid or fluid is the tendency of the liquid molecules to attract each other. Due to the network of hydrogen bonds water has a high surface tension (72 mN/m). Solubilization of ingredients like pectin, polysorbate, and lipophilic essential oil decrease the surface tension of water by disrupting the hydrogen bond network. This means that the tendency of pectin, pectin-polysorbate, and pectin-polysorbate-ZEO particles for attachment to each other was reduced and consequently, particle aggregation was abridged. As a result, the solubilization of pectin-polysorbate-ZEO dispersion to the biological activity increases. The addition of polysorbate-ZEO led to a slight decrease in the viscosity of the pectin particles. Viscosity is a measure of the resistance of a fluid to flow and is related to the internal friction of moving molecules or particles in a fluid. A fluid with low viscosity flows easily because of very little friction between particles during motion. Low-viscosity liquids show little resistance to flow and thus liquid momentum is easily transferred throughout the liquid and low power is required to agitate the liquid. Furthermore, as the viscosity decreases, the diffusion and permeability of the fluid usually increase. ZEO reduced the viscosity of pectin which may be due to the efficient emulsification properties of ZEO. This means at lower temperatures pectin-polysorbate-ZEO dispersion remains more liquefied.

**TABLE 1:** Zeta-potential (mV), particle sizes (nm), surface tension (mN/m), and viscosity (mPa^.^s, at a shear rate of 10 s^−1^) of apple pectin incorporated with Zataria essential oil (ZEO emulsion).

Dispersions	Polysorbate	Polysorbate-ZEO	Pectin	Pectin-polysorbate	Pectin-polysorbate-ZEO
Zeta-potential	4.5 ± 0.85^a^	3.60 ± 0.73^a^	35 ± 2.7^c^	30 ± 2.5^bc^	27 ± 2.0^b^
Particle size	174 ± 6.50^a^	180 ± 8.50^a^	220 ± 7.5^c^	217 ± 7.0^b^	184 ± 6.0^a^
Surface tension	1.55 ± 0.54^b^	1.50 ± 0.47^a^	25 ± 2.0^d^	23 ± 2.0^d^	20 ± 1.7^c^
Viscosity	2.33 ± 0.75^b^	1.78 ± 0.70^a^	37 ± 2.5^e^	33 ± 1.7^d^	25 ± 1.5^c^
The values are expressed as means ± standard deviation. Mean values with different letters within a column are significantly different by Turkey’s test (*p* < 0.05).					

**FIGURE 1:**
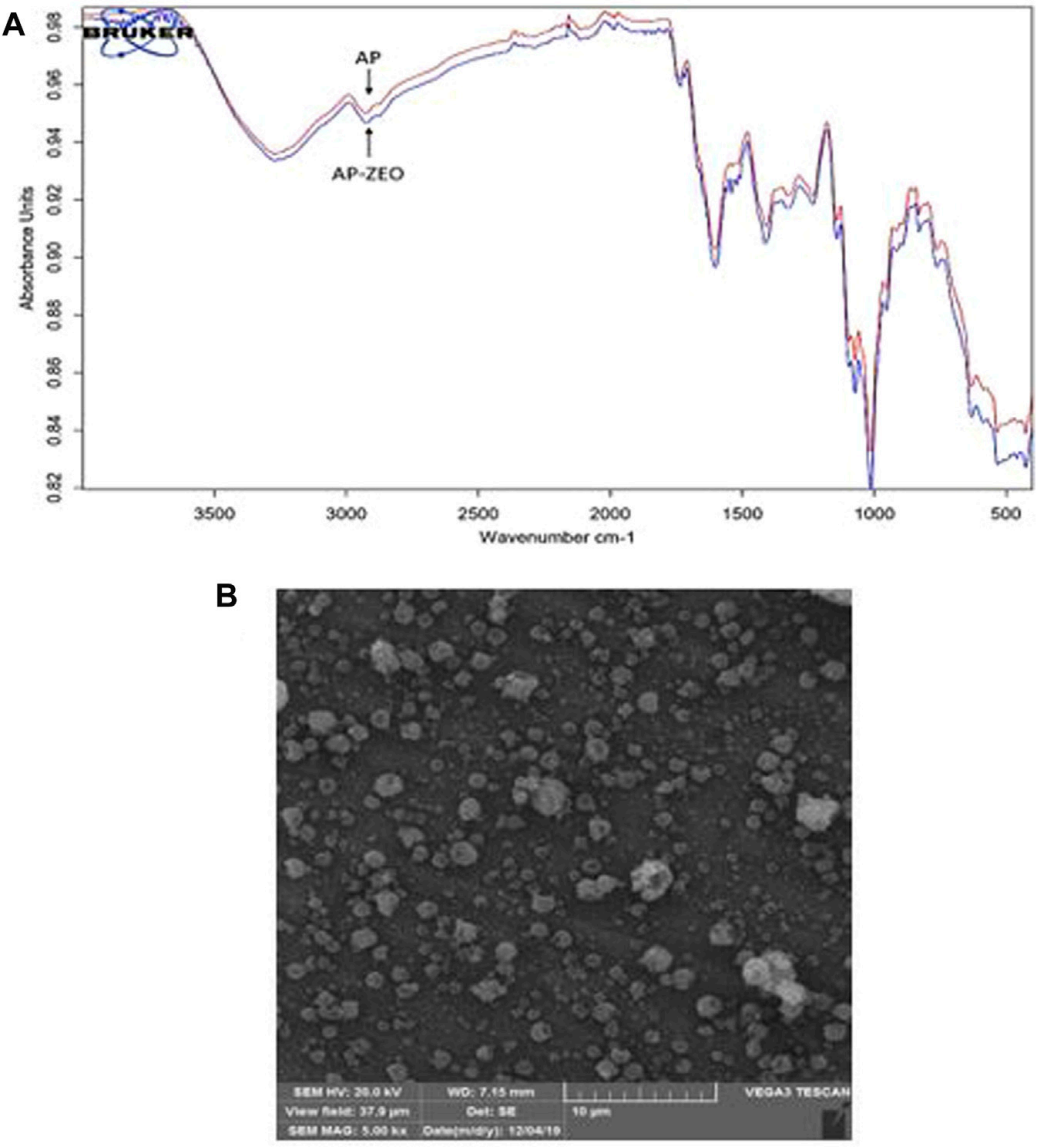
**(A)** FTIR spectra of pectin-polysorbate (AP) AP and AP-ZEONE. High similarity between spectra suggests a non-covalent interaction between AP, polysorbate, and ZEO **(B)** Scanning Electron Microscopy (SEM) image of AP-ZEONE nanoparticles formed on the aluminum sheet by electrospraying.

FTIR spectroscopy of AP revealed alterations in pectin emulsions following combination with ZEO. Pure pectin FTIR spectra were similar to AP-ZEO and reflected the non-covalent interactions between AP and ZEO. Accordingly, FTIR spectral measurement revealed that the bands at 3,700–3,000 cm^−1^ represented the properties of hydrated components attributed to water hydroxyl group stretching vibration and bending. These peaks show the existence of water in pectin particles. The band at 2,939 cm^−1^ was reflected from C-H stretching vibration. The peaks at positions 2,359 cm^−1^ and 2,341 cm^−1^ reflect stretching vibrations of C=C bonds. The band at 2,115 cm^−1^ was attributed to the C=O bond of Carboxylic acid/esters. The peak at position 1,670 is denoted by the C-O bond of Carboxylic acid and esters. The bands 1,589 cm^−1^ and 1,416 cm^−1^ are determined by the C-O stretch of Alcohol. The bands at positions 1,372 cm^−1^, 1,146 cm^−1^, and 1,012 cm^−1^ corresponded to the C-N stretching of Aliphatic amines. The peaks at 1,224 cm^−1^ revealed the C-H stretch of Alkyl. The bands at 668 and 529 cm^−1^ were attributed to C-C bending in alkanes (Figure 1A).

SEM image of the electrosprayed AP-ZEONE nanoparticles exhibited evenly distributed 100–200 nm spheroids (Figure 1B). SEM data is in accordance with particle sizes obtained from DLS analysis (Table 1).

### 3.2 AP-ZEONE cytotoxicity

**FIGURE 2:**
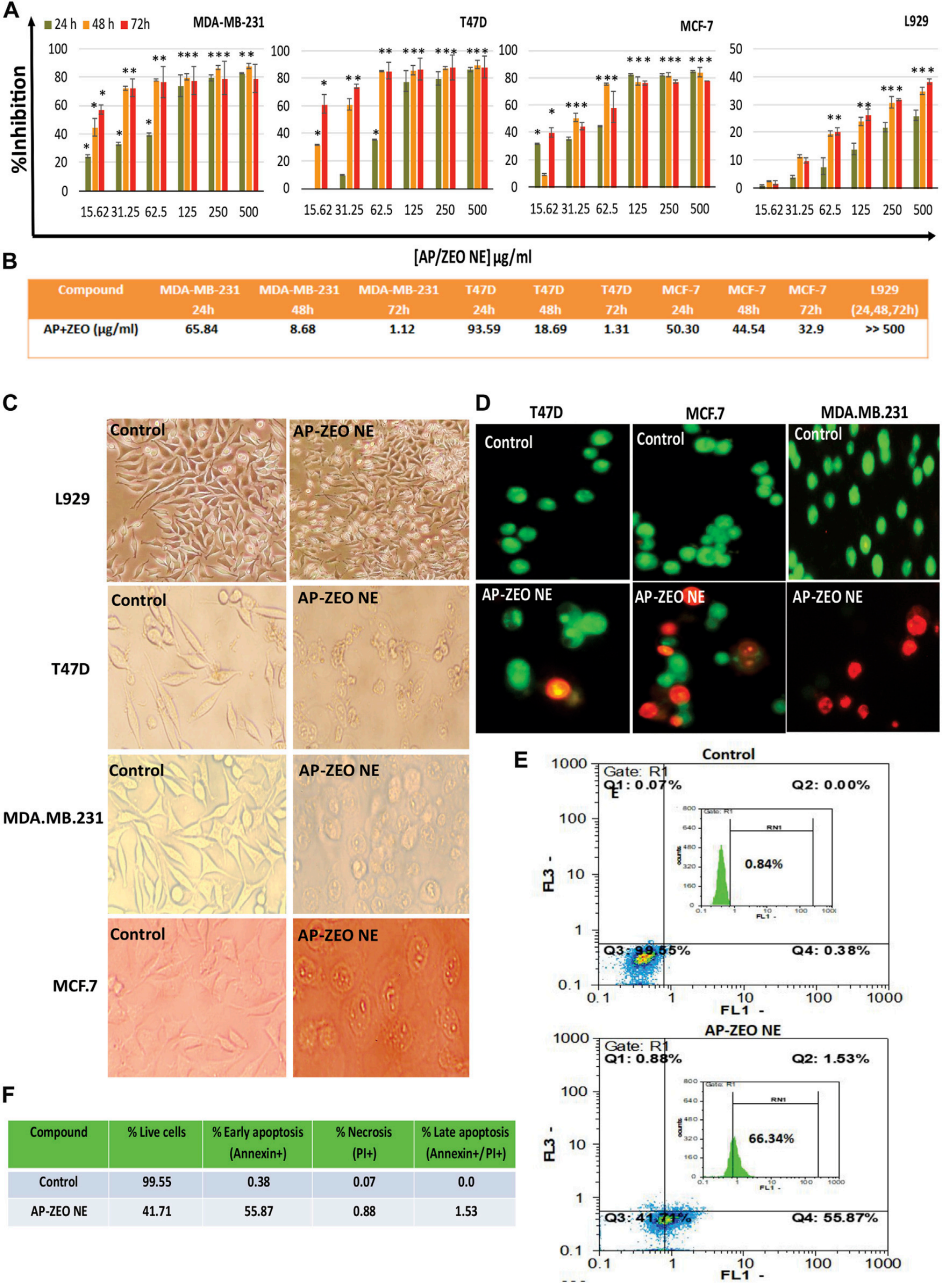
**(A,B)** Cytotoxicity and IC50 (µg/ml) values of AP-ZEONE against T47D, MCF-7, MDA. MB.231, and L929 cell lines. The treatment times were 24, 48, and 72 h, respectively. Apoptotic cells were observed **(C)** as rounded and non-adherent entities, **(D)** blebbing and DNA fragmentation **(E,F)** phosphatidylserine externalization at AP-ZEONE IC50 compared to control intact cells. The “*** “, “** “and “* “indicate the level of statistical significance *p* < 0.001, *p* < 0.01 and *p* < 0.05, respectively.

The tumor cytotoxicity of AP-ZEONE was studied in three human breast cancer cells and a normal cell line for 24, 48, and 72 h by MTT assay. As displayed in Figure 2, AP-ZEONE significantly suppressed the growth of T47D, MCF-7, and MDA. MB.231 cells in a time and concentration-dependent manner. It was found that AP-ZEONE had moderate toxicity against all breast cancer cell lines after 24 h however, its IC50 in MDA-MB-231, MCF-7, and T47D cells decreased to 1.12, 1.31, and 32.9 μg/ml (72 h) respectively, suggesting that AP decreased IC50 of ZEO in breast cancer cells and the Nanoemulsification of ZEO could improve cytotoxicity against breast cancer cells. It is important to note that one of the major obstacles to cancer treatment is the selective killing of cancer cells with minimal damage to normal cells. According to the IC50 values shown in Figure 2, AP-ZEONE also has selectivity toward breast cancer cells and is less toxic than normal cells. The cytotoxicity of AP-ZEONE on L929 cells was negligible at 0–500 μg/ml concentrations and more than 60% of the cells were viable at the maximum concentration of AP-ZEONE (500 μg/ml), suggesting that the AP-ZEONE targeted breast cancer cells and showed no significant cytotoxicity against normal cells. Our results showed that AP-ZEONE exhibited the highest antiproliferative activity on MDA-MB-231 cells after 72 h with an IC50 of 1.12 μg/ml. Moreover, the high cytotoxicity against the MDA-MB-231 cells indicated that AP-ZEONE overcame drug-resistant breast cancer cells. We found that using AP polymer nanoparticles for triggered ZEO release had inhibited multi-drug resistant breast cancer cells more efficiently than the ZEO alone at the same concentration and could induce significant antitumor activity.

### 3.3 Cell death mode characterization

To estimate cellular toxicity at the morphological level, fluorescent and visible light microscopy were implemented to observe morphological changes of AP-ZEONE treated cells (Figure 2C &D). The fusiform-shaped control cells were attached to the surface of the plate. Conversely, there were a large number of non-adherent and rounded cells, when AP-ZEONE was present, suggesting high cytotoxicity. Distinct morphological variations in cancer cells were observed by AO/EB staining (Figure 2D). Evidently, the untreated cells did not display any apoptotic features, while the cells exposed to AP-ZEONE showed progressive apoptotic morphological changes. Microscopic observations showed that AP-ZEONE treatment has induced cellular swelling, rupture of the plasma membrane, nuclear fragmentation, and cell shrinkage which are features of necrotic and apoptotic cells. The cell morphological alterations were in agreement with the quantitative analysis made by the MTT assay, showing that AP-ZEONE displayed a more efficient apoptotic activity in MDA-MB-231 cells. In addition, the Annexin V/PI labeling was performed to study the initial apoptosis by phosphatidylserine externalization to the cell surface. Most Annexin V-PI stained MDA-MB-231 cells were positive against Annexin V.

**FIGURE 3:**
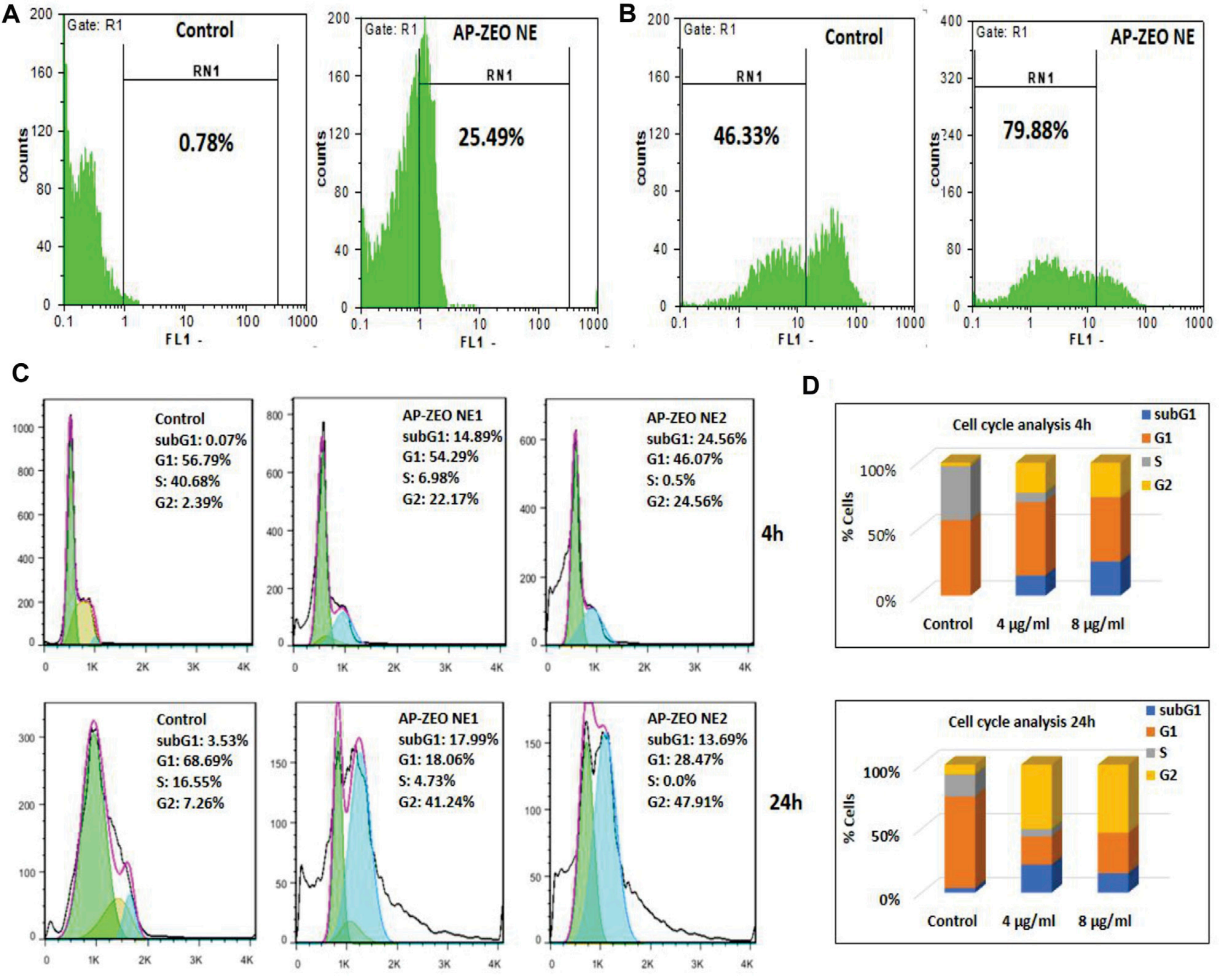
**(A)** Overproduction of ROS by AP-ZEONE **(B)** leading to the loss of MMP. **(C,D)** Cell cycle distributions and percentage of MDA-MB-231 cells in different phases.

The overproduction of ROS acts as a basic chemical signal to trigger apoptosis in cells. In general, the extra amount of intracellular ROS can destroy the balance of redox in cells by attacking various biological molecules, causing DNA damage/fragmentation and inducing cell apoptosis through downstream signaling pathways. Herein, the ROS level in MDA-MB-231 cells exposed to AP-ZEONE was measured by DCFH-DA. We found that the treatment of MDA-MB-231 cell lines with AP-ZEONE triggers intracellular ROS production and acts as a ROS production enhancer in MDA-MB-231 cells (Figure 3A). ROS produced in mitochondria can lead to mitochondrial dysfunction, which is usually associated with loss of mitochondrial membrane potential. MMP alterations induced by the AP-ZEONE complex were detected by flow cytometry with the aid of rhodamin123 dye. In apoptotic cells, Rh123 depolarized monomers release green fluorescence (left peak) while in healthy cells, Rh123 aggregates emit red fluorescence (right peak) via polarization in response to the loss of MMP. After treatment with AP-ZEONE, a significant shift to the left was observed which is due to the significant decrease in MMP. The percentage of cells with depolarized mitochondrial membranes increased from 46.33% (control) to 79.88% (Figure 3B) in AP-ZEONE treated cells. The capability of the AP-ZEONE complex to induce MMP loss is associated with ROS generation. Most anti-cancer drugs arrest the cell cycle in G1, S, or G2/M phases depending on their action. G2 checkpoints are involved in controlling the cell cycle in response to DNA damage and the entrance to mitosis. To detect the changes in the cell cycle, cell cycle perturbation analysis was performed by flow cytometry after treatment of MDA-MB-231 cells with AP-ZEONE. A significant decrease in the G1 fraction (68.69% in control vs 18.06 and 28.47% in 4 and 8 μg/ml treated cells, respectively) and an increase in the G2/M fraction (7.26% in control vs 41.24%, and 47.91% in 4 and 8 μg/ml treated cells, respectively) of the cell population was observed after 24 h treatment (Figure 3C). Based on Figure 3D, it can be suggested that upon treatment with AP-ZEONE, cancer cells exhibit *in vitro* mitotic arrest and chromosomal instability related to the expression profile of the G2/M phase.

### 3.4 AP-ZEONE genotoxicity characterization

**FIGURE 4:**
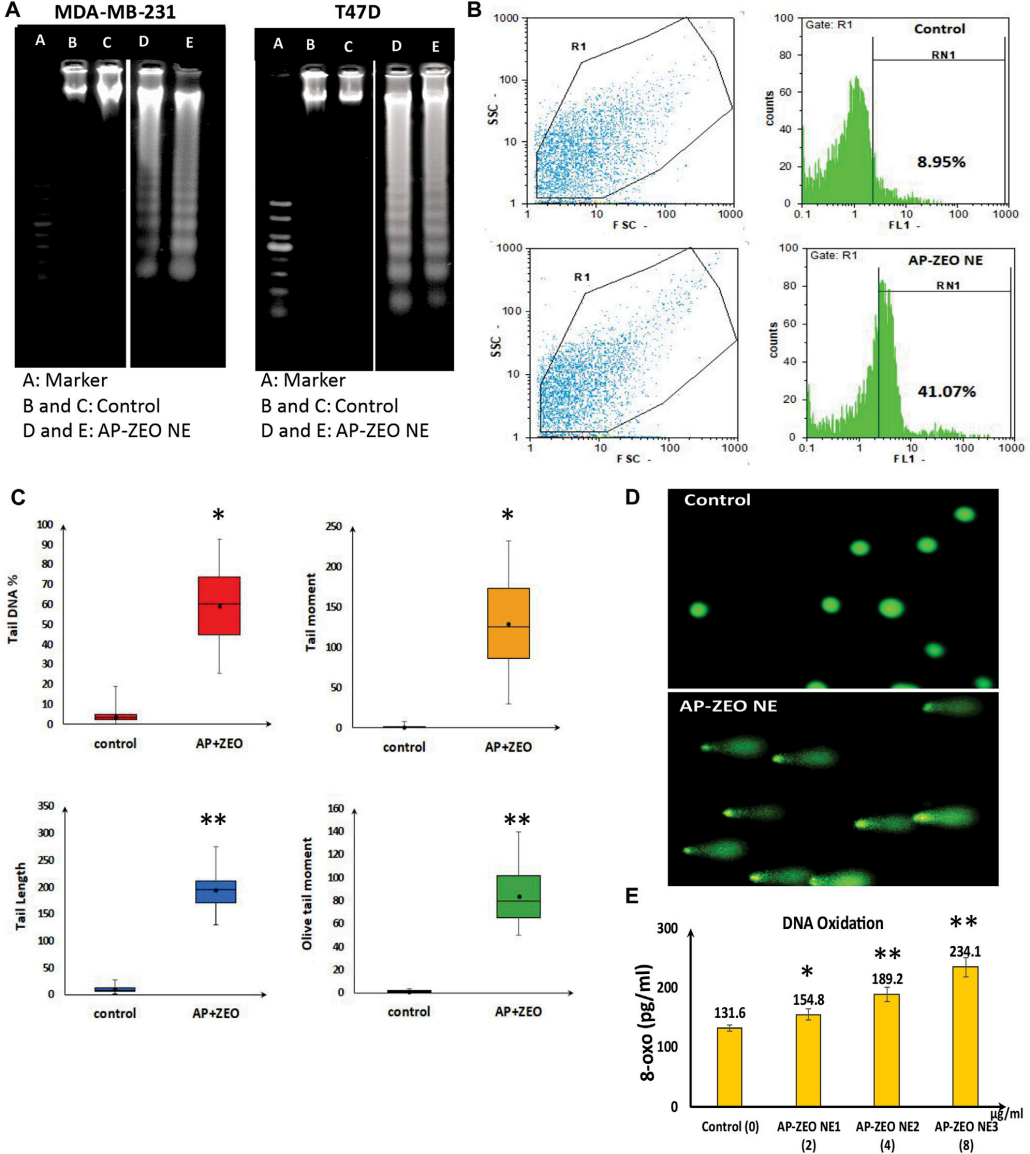
AP-ZEONE induced apoptotic DNA fragmentation **(A)** analysis on agarose gel (Note that this figure was composed of different (cropped) parts of the same gel, indicated by the dividing lines) and **(B)** flow cytometry analysis by TUNEL assay. AP-ZEONE induced Single-cell DNA damage **(C)** as visualized in comet assay and **(D)** analysis performed on Open Comet software. **(E)** Genomic DNA 8-oxo content as a marker of DNA oxidation in AP-ZEONE treated MDA-MB-231 cells and untreated control.”**“ and “*“indicate the level of statistical significance *p* < 0.01 and *p* < 0.05, respectively.

Analysis of cell cycle arrest, Annexin V/PI staining, and DNA damage analysis all together confirmed the apoptosis induction in AP-ZEONE cells. DNA laddering is a kind of DNA damage seen in the late stages of apoptosis, and TUNEL *in situ* apoptosis assay also reveals apoptosis-related DNA fragmentation. As it is shown in Figure 4A, upon treatment with AP-ZEONE, T47D and MDA-MB-231 cells exhibited significant DNA laddering patterns. Moreover, increased TUNEL-positive cells (41.07%) were apparent in MDA-MB-231 cells after treatment with AP-ZEONE IC50 (Figure 4B). To confirm our observation furthermore, single-cell gel electrophoresis “Comet assay” was performed and the DNA damage was quantified at the single-cell level. The fluorescent images of the cell comets took by fluorescence microscope are shown in Figure 4C. The images were analyzed using the openComet plugin in the ImageJ software. These analyses provide an array of data and measure DNA migration. Indeed, DNA damage was quantified and four common parameters such as % tail DNA, tail length, olive tail moment, and tail moment were calculated (Figure 4D). DNA oxidation analysis by Elisa also revealed that by treating MDA-MB-231 cells with increasing amounts of AP-ZEONE the level of oxidative base lesion particularly 8-oxo increases in the treated cells' genomic DNA which can be the result of increased ROS concentration (Figure 4E). Our results showed that AP-ZEONE induces DNA fragmentation, double-strand breaks (DSB), and ROS generation, and increases the number of PI and Annexin V positive cells, which triggers apoptotic morphology.

### 3.5 DNA groove binding interaction of AP-ZEONE

**FIGURE 5:**
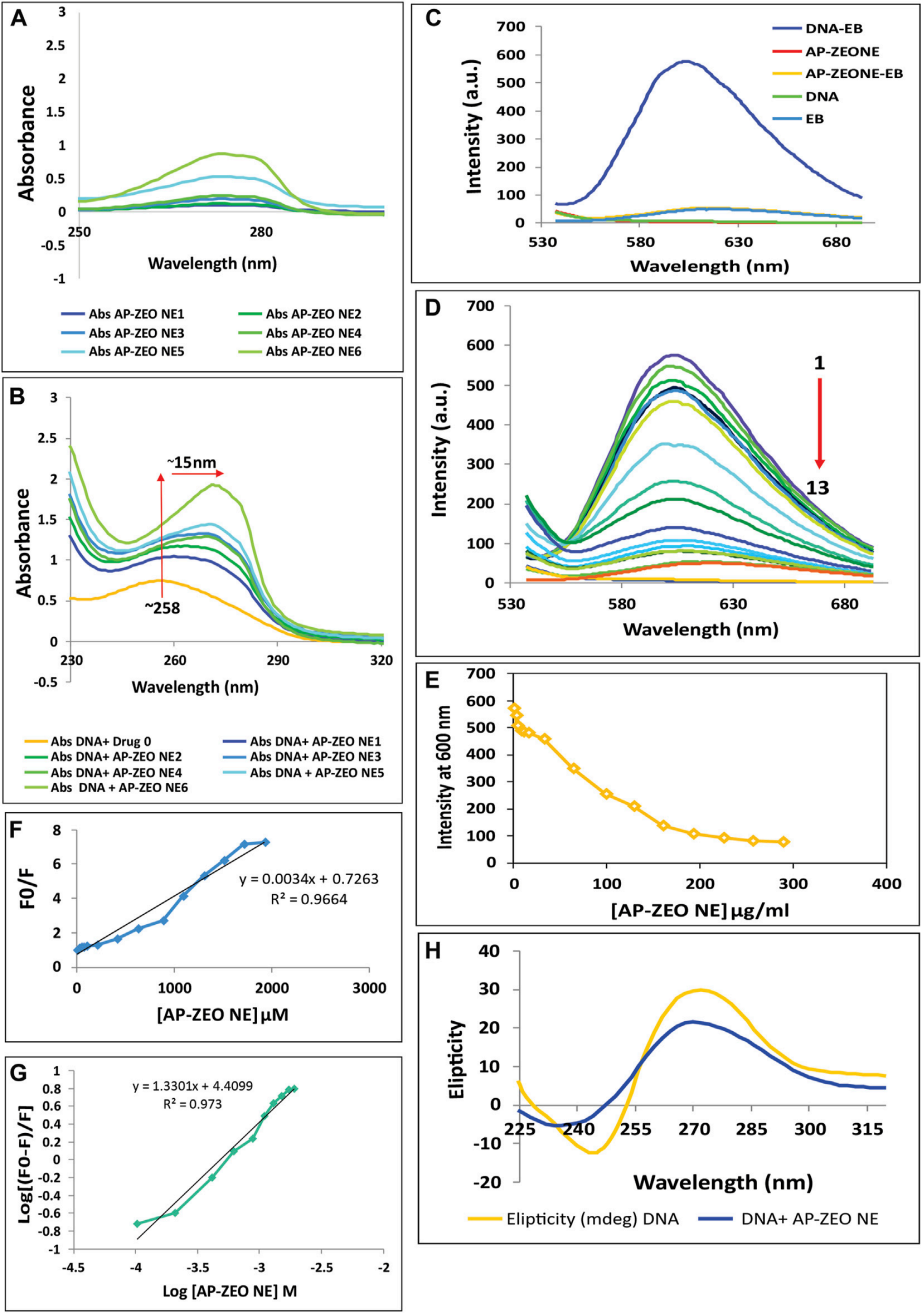
Interaction of AP-ZEONE with RH-DNA **(A)** UV–visible spectra of AP-ZEONE alone **(B)** and AP-ZEONE-DNA complex in the presence of AP-ZEONE (0–32 μg/ml). Hyperchromism with a 15 nm shift was observed with an increasing concentration of AP-ZEONE confirming the interaction of AP-ZEONE and DNA **(C)** Fluorescence spectra of DNA, EB, AP-ZEONE, AP-ZEONE-EB, and DNA-EB. **(D–E)** Competitive displacement assays of DNA-EB in the presence of AP-ZEONE (0–192 μg/ml). The arrow shows the decrease in the fluorescence intensity upon increasing the concentration of AP-ZEONE **(F)** Fluorescence quenching Stern–Volmer plot of DNA-EB and **(G)** Modified Stern–Volmer plot for the quenching of DNA-EB with increasing concentration AP-ZEONE **(H)** The CD spectra of DNA (100 μg/ml) in the presence of an 8 μg/ml concentration of AP-ZEONE.

DNA fragmentation and DSB showed the role of AP-ZEONE in inducing damage to DNA double-helical structure by high oxidative stress in treated cells compared to the controls. To clarify whether AP-ZEONE directly triggers DNA damage, different studies such as CD spectral analysis, and spectroscopic studies based on DNA interaction were performed. In this study, we have focused on deciphering the interaction of RH-DNA with AP-ZEONE by spectroscopic approaches to understand the mechanism of binding. UV–visible spectroscopy as a basic approach was performed to study the complex formation. The UV spectra of AP-ZEONE alone and RH-DNA in the absence or presence of various concentrations of AP-ZEONE are given in Figures 5A,B. The UV spectrum of RH-DNA presented a typical absorption peak at 260 nm. With the progressive addition of AP-ZEONE, a measurable increase in the absorption (hyperchromic shift) of RH-DNA with a significant red shift (∼15 nm) in the maximum absorption position was observed. When a ligand is intercalated into the base pairs of the DNA molecule, the coupling between the former orbital π-antibonding occurs with the latter orbital π-bonding, which reduces the π–π∗ transition energy and subsequent red shift of the absorption region. It is commonly observed that the UV absorption spectra in the classical intercalators exhibit a significant redshift. Hence, the results of absorption spectroscopy suggest that the binding of AP-ZEONE is more likely intercalative, however, determining the binding mode is useless. Further experiments by fluorescence quenching mechanism and CD spectroscopy could reveal the binding mode and present more detailed information. Both AP-ZEONE and DNA have no or very weak fluorescence thus EB is expected to help in determining the interaction of AP-ZEONE with DNA as a fluorescence probe. EB is an intercalator binding agent for DNA and as seen in Figure 5C, the intrinsic fluorescence of EB in the phosphate buffer is very low. However, with the addition of EB to the DNA solution, the fluorescence intensity of the mixture was very strong compared to DNA or EB itself. The planar aromatic chromophore of EB is able to intercalate to the adjacent base pairs on the DNA double helix and form a DNA–EB complex. Therefore, EB was used in this study to investigate the mechanism of AP-ZEONE interaction with DNA. The appropriate concentration of the EB probe was essential for the study. By keeping the DNA (50 μg/ml) and EB (2.6 µM) at constant concentrations and changing the AP-ZEONE concentration, the fluorescence quenching experiments of a series of assay solutions were performed at 300 K (temp.) (Figure 5D). The fluorescence intensity of DNA–EB was regularly decreased when AP-ZEONE was added to the solution containing DNA‒EB but no shift occurred in the peak position of DNA‒EB with the increasing concentration of AP-ZEONE (Figure 5E). The data were analyzed according to the Stern–Volmer equation:

(F0/F)=1+KSV[Q]. (1)

F0 and F represent the fluorescence intensities of DNA-EB before and after the addition of AP-ZEONE (quencher), respectively. [Q] is the concentration of the AP-ZEONE and KSV is the Stern–Volmer constant. The Stern–Volmer plots shown in Figure 5F are linear and the values of KSV for the AP-ZEONE-DNA-EB system were measured. The KSV value for the DNA-AP-ZEONE interaction was 3.4 × 10^9^ M^−1^. This value implies that there is a strong interaction between DNA and AP-ZEONE, which causes the fluorescence intensity quenching with a dynamic pattern. In the comparison of the KSV values with another intercalator, this interaction was consistent with intercalative binding mode. Thus, AP-ZEONE is recommended to interact with DNA in an interactive mode. We calculated the binding constant (Ka) and the number of binding sites (n) by the following modified Stern–Volmer equation:

Log(F0-F)/F=logKa+nlog[Q]. (2)

The n value was equal to 1.33. The value of *K*a ranging from 10^4^ to 10^6^ M^−1^ indicates a strong binding affinity, which suggests that the binding between AP-ZEONE and DNA (*K*a = 2.5×10^4^ M^−1^) could be classified as a moderately strong one (Figure 5G). Therefore, the combination of AP-ZEONE to DNA will occur which indicates that the binding mode is intercalation. CD spectral technique is used to identify even minor conformational changes in the secondary structure of DNA induced by small agents-DNA interactions. The observed CD spectra of right-handed B form RHDNA show a positive (276 nm) and a negative (245 nm) ellipticity, corresponding to base stacking and right-handed helicity, respectively. Groove binding of small agents to DNA shows a decrease in the 276 nm band and an increase in the 245 nm band of CD spectra of DNA. Whereas, a classical intercalator molecule will disturb the DNA helix structure enhancing the intensity of the positive band and decreasing the intensity of the negative band. With increasing AP-ZEONE concentration, the intensity of the positive peak of DNA was decreased and the negative band was increased (Figure 5H), representing that the interaction between AP-ZEONE, and DNA is via groove binding/partial intercalation and does not perturb polynucleotide helicity of DNA.

**FIGURE 6:**
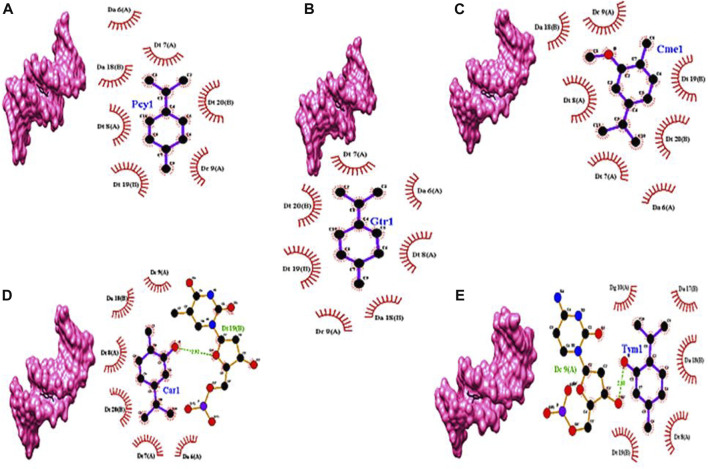
Docking models of **(A)** p-cymene (Pcy1), **(B)** γ-terpinene (Gtr1), **(C)** carvacrol methyl ether (Cme1), **(D)** carvacrol (Car1), and **(E)** thymol (Tym1) with DNA double-helix (PDB ID: 1BNA) represented in Chimera software and 2D presentation of the docked models of in Ligplot^+^. Residues of the DNA molecule involved in hydrogen bonds and hydrophobic interactions with the chemicals are represented by the dashed green lines and brick-red arcs, respectively.

Docking studies of carvacrol, γ-terpinene, carvacrol methyl ether, p-cymene, and thymol against genomic DNA indicated the interaction via minor groove of DNA molecule (Figure 6). This result signifies the interaction of ZEO with DNA molecules.

## 4 Discussion

Plants' secondary metabolites have attracted interest in cancer treatment due to their effectiveness and low toxicity. These natural compounds are able to manipulate signaling pathways to alter cell proliferation or induce apoptosis, etc. For instance, phenolic-rich phytochemicals induce high growth inhibition in colon, breast, prostate, and cervical cancer cells (Mazewski et al., 2018; Singh et al., 2018). ZEO similar to other essential oils is lipophilic in nature and can readily penetrate the cell membrane to reach inside the cell. In a previous study, GC/MS analysis of ZEO revealed Carvacrol, *γ*-terpinene, Carvacrol methyl ether, *p*-cymene, and Thymol as its main components, respectively (Salehi et al., 2017). On the other hand, *Oliveria decumbens* Essential oil (OEO) like ZEO has a similar phenolic compounds profile and it was shown to have anticancer activity against 4T1 cell line *in vitro* and 4T1 tumor mouse model *in vivo* (Jamali et al., 2020)*.* In another study, the anti-proliferative effect of three major components of OEO, Carvacrol and *p*-cymene and Thymol, were investigated. The results demonstrated that Carvacrol and Thymol can obviously cause the growth inhibition of MDA-MB231 and MCF-7 cells while *p*-cymene has no significant effect on cancer cell proliferation (Jamali et al., 2018). Thymol and Carvacrol have also been found to possess some anti-cancer activity in cancer cell culture, but their underlying mechanism has not yet been fully elucidated (Coccimiglio et al., 2016; Khan et al., 2018). Moreover, our *in silico* simulations also showed that Carvacrol and Thymol have more robust interaction with DNA compared to *p*-cymene. It seems that the cytotoxic effect of ZEO is due to its multi-component nature and synergistic effect of multiple compounds such as Thymol and Carvacrol. Due to essential oils' degradability and volatile nature in order to optimize their pharmaceutical performance and stability multiple drug delivery systems have been improvised. Nano emulsification of phytochemicals by natural compounds is a novel and safe drug delivery approach to cancer cells (Navaei Shoorvarzi et al., 2020; Abadi et al., 2022). In this study, we produced solid spherical nanoemulsions containing ZEO ranging from 100–184 nm via the non-covalent interactions between AP and ZEO. The nanometric size of the droplets in essential oils nanoemulsions improves their bioavailability by increasing their absorbency, permeability, and stability (Javanshir et al., 2020; Bashlouei et al., 2022). The growth inhibitory effect of AP-ZEONE on T47D, MCF-7, and MDA-MB-231 cells without any toxicity on L929 was concluded from the MTT assay, showed the selectivity effect of AP-ZEONE on cancer cells and strengthened the suitability of AP-ZEONE as an effective agent. Since cell death induced by AP-ZEONE resembled to have mediated by triggering the apoptotic pathways, in the following, phosphatidylserine exposure on the surface of the AP-ZEONE treated MDA-MB-231 cells was detected by annexin-V-FITC flow cytometry assay. In addition, the DNA fragmentation assay vividly exhibited the DNA laddering pattern specific to apoptotic cells. The level of oxidative stress is generally high in cancer cells. Although, this property is one of the promoters of cancer development, however, it makes cancer cells susceptible to further ROS levels (Yang et al., 2013; Tong et al., 2015). Several anti-cancer drugs induce apoptosis in cancer cells by increasing ROS levels, however, the underlying mechanism is not clear (Ivanova et al., 2016). ROS-induced oxidative stress induced by ROS can directly or through DNA damage and cell cycle arrest, hyperpolarization of ΔΨm, and cytochrome C discharge provoke apoptosis (Redza-Dutordoir and Averill-Bates, 2016). Our study revealed that cancer cells treated with AP-ZEONE increase ROS levels. One of the important targets of ROS is the guanine nucleobases in DNA and RNA structure. 8-OxodG is the main DNA adduct generated by oxidative damage. The existence of 8-oxodG in the genomic DNA is very mutagenic and provokes apoptosis (Rai et al., 2011). Mitochondria membrane depolarization also increases the ROS level, elevated ROS levels on the other hand cause the loss of mitochondrial membrane potential (ΔΨm). In fact, during early apoptosis, mitochondrial dysfunction is an important phenomenon that triggers caspase activity and finally the intrinsic pathway of apoptosis. Our findings showed that AP-ZEONE is able to decrease MMP and thus it suggested that AP-ZEONE induces the apoptotic mitochondrial pathway in AP-ZEONE treated MDA-MB-231 cell lines. It is worth mentioning that in this study, very low concentrations of AP-ZEONE were used, and thus AP-ZEONE is not able to behave like a pro-oxidant. Indeed, according to previous studies, ZEO has a high antioxidant capacity and can scavenge active oxygen and nitrogen radicals (Karimian et al., 2012; Kavoosi et al., 2012). Therefore, AP-ZEONE not only does not produce active oxygen and nitrogen radicals such as hydrogen peroxide, but it is also capable of eliminating excess radicals in the culture medium. Therefore, the effect of AP-ZEONE on cancer cells is a direct effect resulting from permeability in the cell. Induction of cell cycle arrest to prevent cell proliferation is one of the effective mechanisms used by cytotoxic agents. Treatment of MDA-MB231 with various concentrations of AP-ZEONE clearly showed that AP-ZEONE has the potential to arrest MDA-MB231 cell lines at the G2/m-phase of the cell cycle significantly and trigger apoptosis in treated cancer cells. Another valuable feature of the anticancer agent is its capability to interact with DNA. DNA structure, as the major cellular pharmacological target for the cytotoxic activity of antitumor drugs, can be destroyed upon binding and interacting with various small molecules. Studying such interactions has attracted wide attention and provided evidence of the functioning of these drugs (Pawar et al., 2019). Most anti-cancer drugs work by producing ROS and damaging DNA (Mallick et al., 2016; Sau et al., 2018). The diverse interaction between these drugs and DNA is indeed a promising area to be explored. One initial goal in monitoring DNA-drug interaction is to probe the binding mode of the drug to DNA. Drugs may bind to the DNA in a covalent or noncovalent mode. Non-covalent binding mode occurs through minor and/or major groove, intercalative and electrostatic binding (Jana et al., 2012). Indeed, DNA interaction alters the cell's fate by replication inhibition and/or transcription alteration. Minor groove-binding molecules often bind DNA non-covalently at A/T-rich regions and have the potential to interfere with specific proteins binding in these regions. Furthermore, some of these agents inhibit the action of topoisomerases which are required mainly at the time of DNA replication and transcription (Cai et al., 2009). For instance, distamycin A and pentamidine, bind to the DNA minor groove and form non-covalent complexes (Cai et al., 2009). Spectroscopic studies, CD spectral analysis, and *in-silico* molecular docking revealed that the addition of AP-ZEONE to dsDNA solutions shifts UV-Vis spectra of DNA to hyperchromicity which confirms non-covalent interactions in DNA- AP-ZEONE complexes. Moreover, EB competitive displacement assays showed that AP-ZEONE intercalates into the DNA base pairs. These data were completed by CD spectral analysis that shows groove binding mode of interaction. On the other hand, In *In-silico* molecular docking of the five main components of ZEO (Thymol, Carvacrol, Carvacrol methyl ether, *p*-cymene, and γ-terpinene) showed that Carvacrol and Thymol can bind to the DNA minor groove at thymidine and cytosine rich sequences with satisfactory binding energy, respectively, while *p*-cymene, Carvacrol methyl ether and γ-terpinene bind to the minor groove with lower binding affinity. Briefly, these findings showed that AP-ZEONE imposes its effect on cancer cells through interaction with DNA. Indeed, with the creation of a complex between DNA and AP-ZEONE, DNA replication is probably disrupted and cell cycle arrest occurs. In addition, binding of AP-ZEONE to DNA may affect the transcription activities inside the cells and modify gene expression leading to altered regulation of cell proliferation and finally cell death.

## 5 Conclusion

Taken together, AP-ZEONE treatment stimulates DNA fragmentation that leads to apoptosis and necrosis-related morphological changes. Moreover, AP-ZEONE exhibited enhanced cytotoxicity, apoptosis induction, and G2/M cell cycle arrest in the drug-resistant MDA-MB-231 cells. Additionally, AP-ZEONE could trigger more apoptosis-related ROS production, MMP loss, and DSB induction in MDA-MB-231 breast cancer cells. AP-ZEONE demonstrated excellent selectivity in the killing of drug-resistant MDA-MB-231 cells without any harmful effect on normal cells. In addition, we investigated the interaction of AP-ZEONE with DNA using different spectroscopic and molecular modeling techniques. The analysis of fluorescence quenching studies and UV absorption showed the formation of a complex between AP-ZEONE and DNA. The quenching of the fluorescence emission spectrum of AP-ZEONE and hyperchromic effect with redshift indicated that it binds DNA probably in an interactive mode. CD study further showed that the interaction of AP-ZEONE with DNA was through a groove binding mode. These data were clarified by computational studies. The five main compounds of ZEO interact with DNA in a minor groove binding mode. Our results proved that the use of nanoemulsion formulation of ZEO, which provides stability and high efficacy, significantly enhances antitumor activity, conceivably more promising for breast cancer therapy. Moreover, among different routes that essential oils can be administered, our AP-ZEONE is suitable as edible syrup. Biochemical pathways involved in the apoptosis activity of AP-ZEONE are needed to be investigated further. Moreover, the effectiveness of this new formulation should be tested *in vivo*.

## Data Availability

The original contributions presented in the study are included in the article/Supplementary Material; further inquiries can be directed to the corresponding author.

## References

[B1] Abadi A. V. M. Karimi E. Oskoueian E. Mohammad G. R. K. S. Shafaei N. (2022). Chemical investigation and screening of anti-cancer potential of Syzygium aromaticum L. bud (clove) essential oil nanoemulsion. 3 Biotech. 12, 49–10. 10.1007/s13205-022-03117-2 PMC8795257 35127304

[B2] Ainsworth E. A. Gillespie K. M. (2007). Estimation of total phenolic content and other oxidation substrates in plant tissues using Folin–Ciocalteu reagent. Nat. Protoc. 2, 875–877. 10.1038/nprot.2007.102 17446889

[B3] Ali H. Al-Khalifa A. R. Aouf A. Boukhebti H. Farouk A. (2020). Effect of nanoencapsulation on volatile constituents, and antioxidant and anticancer activities of algerian origanum glandulosum Desf. essential oil. Sci. Rep. 10, 2812–2819. 10.1038/s41598-020-59686-w 32071359 PMC7028938

[B4] Bashlouei S. G. Karimi E. Zareian M. Oskoueian E. Shakeri M. (2022). Heracleum persicum essential oil nanoemulsion: a nanocarrier system for the delivery of promising anticancer and antioxidant bioactive agents. Antioxidants 11, 831. 10.3390/antiox11050831 35624695 PMC9138159

[B5] Cai X. Gray P. J. Jr Von Hoff D. D. (2009). DNA minor groove binders: back in the groove. Cancer Treat. Rev. 35, 437–450. 10.1016/j.ctrv.2009.02.004 19328629

[B6] Coccimiglio J. Alipour M. Jiang Z.-H. Gottardo C. Suntres Z. (2016). Antioxidant, antibacterial, and cytotoxic activities of the ethanolic origanum vulgare extract and its major constituents. Oxidative Med. Cell. Longev. 2016, 1404505. 10.1155/2016/1404505 PMC4804097 27051475

[B7] Ghasemi Pirbalouti A. Hamedi B. Abdizadeh R. Malekpoor F. (2011). Antifungal activity of the essential oil of Iranian medicinal plants. J. Med. Plants Res. 5 (20), 5089–5093.

[B8] Hati S. Tripathy S. Dutta P. K. Agarwal R. Srinivasan R. Singh A. (2016). Spiro [pyrrolidine-3, 3-oxindole] as potent anti-breast cancer compounds: Their design, synthesis, biological evaluation and cellular target identification. Sci. Rep. 6, 32213–32310. 10.1038/srep32213 27573798 PMC5004205

[B9] Ivanova D. Zhelev Z. Aoki I. Bakalova R. Higashi T. (2016). Overproduction of reactive oxygen species-obligatory or not for induction of apoptosis by anticancer drugs. Chin. J. Cancer Res. 28, 383–396. 10.21147/j.issn.1000-9604.2016.04.01 27647966 PMC5018533

[B10] Jamali T. Kavoosi G. Ardestani S. K. (2020). *In-vitro* and *in-vivo* anti-breast cancer activity of OEO (Oliveria decumbens vent essential oil) through promoting the apoptosis and immunomodulatory effects. J. Ethnopharmacol. 248, 112313. 10.1016/j.jep.2019.112313 31655147

[B11] Jamali T. Kavoosi G. Safavi M. Ardestani S. K. (2018). *In-vitro* evaluation of apoptotic effect of OEO and thymol in 2D and 3D cell cultures and the study of their interaction mode with DNA. Sci. Rep. 8, 15787–15819. 10.1038/s41598-018-34055-w 30361692 PMC6202332

[B12] Jana B. Senapati S. Ghosh D. Bose D. Chattopadhyay N. (2012). Spectroscopic exploration of mode of binding of ctDNA with 3-hydroxyflavone: A contrast to the mode of binding with flavonoids having additional hydroxyl groups. J. Phys. Chem. B 116, 639–645. 10.1021/jp2094824 22128894

[B13] Javanshir A. Karimi E. Maragheh A. D. Tabrizi M. H. (2020). The antioxidant and anticancer potential of Ricinus communis L. essential oil nanoemulsions. Food Meas. 14, 1356–1365. 10.1007/s11694-020-00385-5

[B14] Karimian P. Kavoosi G. Saharkhiz M. J. (2012). Antioxidant, nitric oxide scavenging and malondialdehyde scavenging activities of essential oils from different chemotypes of Zataria multiflora. Nat. Prod. Res. 26, 2144–2147. 10.1080/14786419.2011.631136 22054260

[B15] Kavoosi G. Rabiei F. (2015). Zataria multiflora: chemical and biological diversity in the essential oil. J. Essent. Oil Res. 27, 428–436. 10.1080/10412905.2015.1031917

[B16] Kavoosi G. Teixeira Da Silva J. A. Saharkhiz M. J. (2012). Inhibitory effects of Zataria multiflora essential oil and its main components on nitric oxide and hydrogen peroxide production in lipopolysaccharide-stimulated macrophages. J. Pharm. Pharmacol. 64, 1491–1500. 10.1111/j.2042-7158.2012.01510.x 22943180

[B17] Khan I. Bahuguna A. Kumar P. Bajpai V. K. Kang S. C. (2018). *In vitro* and *in vivo* antitumor potential of carvacrol nanoemulsion against human lung adenocarcinoma A549 cells via mitochondrial mediated apoptosis. Sci. Rep. 8, 144–215. 10.1038/s41598-017-18644-9 29317755 PMC5760660

[B18] Kurtz S. L. Lawson L. B. (2019). Nanoemulsions enhance *in vitro* transpapillary diffusion of model fluorescent dye nile red. Sci. Rep. 9, 11810–11811. 10.1038/s41598-019-48144-x 31413320 PMC6694173

[B19] Mallick A. More P. Syed M. M. K. Basu S. (2016). Nanoparticle-mediated mitochondrial damage induces apoptosis in cancer. ACS Appl. Mat. Interfaces 8, 13218–13231. 10.1021/acsami.6b00263 27160664

[B20] Mann J. (2002). Natural products in cancer chemotherapy: Past, present and future. Nat. Rev. Cancer 2, 143–148. 10.1038/nrc723 12635177

[B21] Mazewski C. Liang K. De Mejia E. G. (2018). Comparison of the effect of chemical composition of anthocyanin-rich plant extracts on colon cancer cell proliferation and their potential mechanism of action using *in vitro*, *in silico*, and biochemical assays. Food Chem. 242, 378–388. 10.1016/j.foodchem.2017.09.086 29037704

[B22] Navaei Shoorvarzi S. Shahraki F. Shafaei N. Karimi E. Oskoueian E. (2020). Citrus aurantium L. bloom essential oil nanoemulsion: Synthesis, characterization, cytotoxicity, and its potential health impacts on mice. J. Food Biochem. 44, e13181. 10.1111/jfbc.13181 32173879

[B23] Pawar S. Tandel R. Kunabevu R. Jaldappagari S. (2019). Spectroscopic and computational approaches to unravel the mode of binding between a isoflavone, biochanin-A and calf thymus DNA. J. Biomol. Struct. Dyn. 37, 846–856. 10.1080/07391102.2018.1442748 29458302

[B24] Rai P. Young J. J. Burton D. G. Giribaldi M. G. Onder T. T. Weinberg R. A. (2011). Enhanced elimination of oxidized guanine nucleotides inhibits oncogenic RAS-induced DNA damage and premature senescence. Oncogene 30, 1489–1496. 10.1038/onc.2010.520 21076467

[B25] Redza-Dutordoir M. Averill-Bates D. A. (2016). Activation of apoptosis signalling pathways by reactive oxygen species. Biochim. Biophys. Acta 1863, 2977–2992. 10.1016/j.bbamcr.2016.09.012 27646922

[B26] Saei-Dehkordi S. S. Tajik H. Moradi M. Khalighi-Sigaroodi F. (2010). Chemical composition of essential oils in zataria multiflora Boiss. from different parts of Iran and their radical scavenging and antimicrobial activity. Food Chem. Toxicol. 48, 1562–1567. 10.1016/j.fct.2010.03.025 20332011

[B27] Sajed H. Sahebkar A. Iranshahi M. (2013). Zataria multiflora boiss.(shirazi thyme)—An ancient condiment with modern pharmaceutical uses. J. Ethnopharmacol. 145, 686–698. 10.1016/j.jep.2012.12.018 23266333

[B28] Salehi F. Behboudi H. Kavoosi G. Ardestani S. K. (2020). Incorporation of zataria multiflora essential oil into chitosan biopolymer nanoparticles: a nanoemulsion based delivery system to improve the *in-vitro* efficacy, stability and anticancer activity of ZEO against breast cancer cells. Int. J. Biol. Macromol. 143, 382–392. 10.1016/j.ijbiomac.2019.12.058 31830446

[B29] Salehi F. Behboudi H. Kavoosi G. Ardestani S. K. (2017). Monitoring ZEO apoptotic potential in 2D and 3D cell cultures and associated spectroscopic evidence on mode of interaction with DNA. Sci. Rep. 7, 2553–2614. 10.1038/s41598-017-02633-z 28566685 PMC5451462

[B30] Sau A. Sanyal S. Bera K. Sen S. Mitra A. K. Pal U. (2018). DNA Damage and apoptosis induction in cancer cells by chemically engineered thiolated riboflavin gold nanoassembly. ACS Appl. Mat. Interfaces 10, 4582–4589. 10.1021/acsami.7b18837 29338178

[B31] Singh B. Singh J. P. Kaur A. Singh N. (2018). Phenolic compounds as beneficial phytochemicals in pomegranate (punica granatum L.) peel: a review. Food Chem. 261, 75–86. 10.1016/j.foodchem.2018.04.039 29739608

[B32] Tong L. Chuang C.-C. Wu S. Zuo L. (2015). Reactive oxygen species in redox cancer therapy. Cancer Lett. 367, 18–25. 10.1016/j.canlet.2015.07.008 26187782

[B33] Yang Y. Karakhanova S. Werner J. V Bazhin A. (2013). Reactive oxygen species in cancer biology and anticancer therapy. Curr. Med. Chem. 20, 3677–3692. 10.2174/0929867311320999165 23862622

